# Integration of transcriptomic and genomic data suggests candidate mechanisms for APOE4-mediated pathogenic action in Alzheimer’s disease

**DOI:** 10.1038/srep32583

**Published:** 2016-09-02

**Authors:** Laura Caberlotto, Luca Marchetti, Mario Lauria, Marco Scotti, Silvia Parolo

**Affiliations:** 1 The Microsoft Research, University of Trento Centre for Computational Systems Biology (COSBI), Piazza Manifattura 1, 38068, Rovereto, Italy; 2 GEOMAR Helmholtz Centre for Ocean Research Kiel, Düsternbrooker Weg 20, 24105 Kiel, Germany

## Abstract

Among the genetic factors known to increase the risk of late onset Alzheimer’s diseases (AD), the presence of the apolipoproteine e4 (APOE4) allele has been recognized as the one with the strongest effect. However, despite decades of research, the pathogenic role of APOE4 in Alzheimer’s disease has not been clearly elucidated yet. In order to investigate the pathogenic action of APOE4, we applied a systems biology approach to the analysis of transcriptomic and genomic data of APOE44 vs. APOE33 allele carriers affected by Alzheimer’s disease. Network analysis combined with a novel technique for biomarker computation allowed the identification of an alteration in aging-associated processes such as inflammation, oxidative stress and metabolic pathways, indicating that APOE4 possibly accelerates pathological processes physiologically induced by aging. Subsequent integration with genomic data indicates that the Notch pathway could be the nodal molecular mechanism altered in APOE44 allele carriers with Alzheimer’s disease. Interestingly, PSEN1 and APP, genes whose mutation are known to be linked to early onset Alzheimer’s disease, are closely linked to this pathway. In conclusion, APOE4 role on inflammation and oxidation through the Notch signaling pathway could be crucial in elucidating the risk factors of Alzheimer’s disease.

Alzheimer’s disease (AD) is the most common cause of dementia, characterized clinically by a decline in cognitive function and by distinctive brain pathology with neuronal loss and the formation of amyloid plaques and neurofibrillary tangles.

Early onset AD is rare and is caused by mutations in specific genes such as amyloid precursor protein (APP), presenilin 1 (PSEN1) and presenilin 2 (PSEN2). Late onset AD is the most common form but, although several putative susceptibility genes have been reported, APOE, coding for the Apolipoprotein E, is the most robust susceptibility gene known to date. Three common isoforms of APOE have been recognized: APOE2 (cys112, cys158), APOE3 (cys112, arg158) and APOE4 (arg112, arg158) and the presence of the alleles coding for the APOE4 isoform are associated with an increased risk (up to tenfold in homozygous cases^1^) of late onset AD when compared to the most common APOE3 allele or APOE2, a rarer allele, that appears to have, instead, a protective effect^2^,^3^.

APOE is a multifunctional glycosylated protein with a major role in lipid transport and atherosclerosis pathogenesis and it is expressed in several organs, with the highest expression in the liver and brain. In the central nervous system, although neurons can produce APOE under certain conditions, non-neuronal cells, mainly astrocytes and to some extent microglia, are the major cell types that express APOE in the brain^4^,^5^.

Numerous mechanisms by which APOE influences AD pathogenesis have been proposed, including a role in the clearance of Amyloid β^6^,^7^, but how this influences the pathogenic molecular processes remains to be clarified.

To gain some insight into the molecular mechanisms responsible for the pathogenic impact of APOE, we used a systems biology approach to integrate pre-existing genomic and transcriptomic data in an unbiased fashion. A network-based analysis combined with a novel technique for biomarker identification revealed specific biological processes altered in APOE44 carriers affected by AD. Molecular processes related to inflammation, oxidative stress and metabolism were shown to be altered, demonstrating that APOE4 possibly quickens pathological alterations naturally induced by aging. The integration of genomic data evidenced that components of the Notch pathway could be among the upstream effectors involved in this action.

Overall, this study provides a contribution to the long-standing APOE4 debate by suggesting an evidence driven hypothesis on the mechanism by which APOE4 confers risk for the development of Alzheimer’s disease.

## Material and Methods

**Figure 1:**
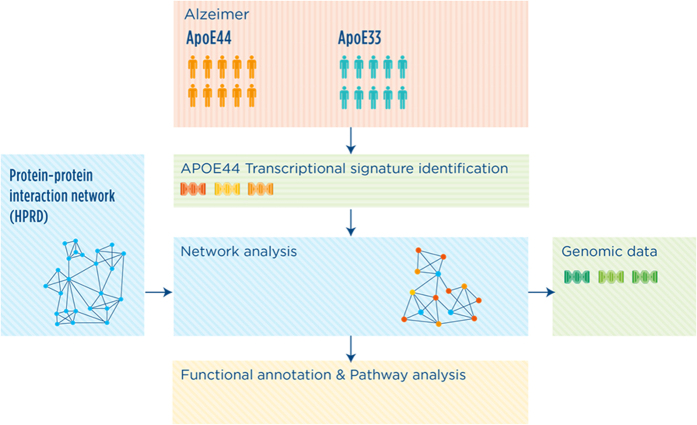
Schematic representation of the network analysis workflow. The molecular biomarker associated to APOE44-AD was extracted from transcriptomic data of post-mortem cerebral cortices of AD affected individuals carrying the APOE33 or APOE44 alleles. Network analysis was then performed using NetWalker technique with the reference protein-protein interaction (PPI) network derived from HPRD data. The functionality of the network was then performed by testing over-represented Gene Ontology biological process terms and pathways. Genomic data analysis was then performed with the analysis of genes in the network displaying a genetic interaction with APOE.

A schematic representation of the study workflow is shown in Fig. 1.

### Transcriptomic and Genomic data

The microarray dataset (study number 1, Table 1) was downloaded from the Myers laboratory website (http://labs.med.miami.edu/myers/LFuN/data.html, GEO reference: GSE15222). The complete dataset consists of transcriptome-wide gene expression data from post-mortem cerebral cortical area (temporal, parietal and frontal cortices), all cortical areas involved in the disease, of Alzheimer’s disease affected subjects at all stages of the disease (Braak stages ≥3) and matched controls (188 controls and 176 AD), stratified by their APOE genotype^8^. In the study, data from 77 subjects affected by AD (43 APOE33 and 34 APOE44) was used (Table 1) matched for age (average age APOE33-AD 83.58 years [72–102 years]; APOE44-AD 83.62 years [68–92 years]) and post-mortem interval (average PMI APOE33-AD 10.54 hours [1.3–26.9 hours]; APOE44-AD 10.39 hours [1.2–17.7 hours]).

For the validation studies, an additional dataset was evaluated: the Mayo RNA sequencing study (study number 2 Table 1) of temporal cortex accessed through the AMP AD Knowledge Portal (syn3163039; https://www.synapse.org/#!Synapse:syn2580853/wiki/77552). The original study consisted of RNA sequencing data of 278 samples from post-mortem temporal cortical tissue of AD affected individuals at all stages of the disease (Braak stages ≥4) and matched controls. In the present study a sub-population of 39 subjects was used (30 APOE33 and 9 APOE44 carriers) all subjects were affected by AD (average age APOE33-AD 76.5 years [65–80 years]; APOE44-AD 72.3 years [66–78 years] (Table 1). No information on post-mortem interval was provided.

In order to evaluate the role of the specific pathways highlighted in our network analysis in astrocytes of APOE4 individuals, an additional dataset was used (GEO accession: GSE29652; study number 3, Table 1). This dataset includes microarray profiles of laser-captured microdissected astrocytes of post-mortem human temporal cortex of AD affected individuals APOE4- and APOE4+ carriers at different Braak stages. NCBI GEO’s tool GEO2R was used to compare expression between the APOE4- and APOE4+ groups, each group including subjects at all stages of the disease. GEO2R analyzes gene expression using GEOquery and the Linear Models of Microarray Analysis R package (limma)^9^,^10^. Genes were considered differentially expressed if they showed a minimum 1.5-fold change at p-value < 0.02 for consistency with the original analysis of dataset GSE29652, as reported in Simpson *et al.*^11^. No correction for multiple testing was performed.

For the genetic analysis, we considered two datasets of genome-wide genotypic data. The first dataset was obtained from the Alzheimer’s Disease Neuroimaging Initiative (ADNI) database (adni.loni.usc.edu; study number 4, Table 1). The ADNI was launched in 2003 as a public-private partnership, led by Principal Investigator Michael W. Weiner, MD. The primary goal of ADNI has been to test whether serial magnetic resonance imaging (MRI), positron emission tomography (PET), other biological markers, and clinical and neuropsychological assessment can be combined to measure the progression of mild cognitive impairment (MCI) and early Alzheimer’s disease. The genotype data of the ADNI dataset were generated using Illumina Human610-Quad BeadChip and referred to 757 individuals. The second dataset was acquired from the Mayo Clinic LOAD Risk Genetic Association study, accessed through the AMP AD Knowledge Portal (syn3157238; study number 5, Table 1). The Mayo LOAD GWAS study originally included 844 cases and 1255 controls, genotyped using the Illumina’s HumanHap300-Duo Genotyping BeadChips. For both dataset we selected the individuals in which the disease status, age of onset and APOE genotype were available.

### Biomarker identification

The transcriptional biomarker has been identified by means of an enhanced version of the rank-based classification method previously implemented in the web-tool SCUDO^12^,^13^. Briefly, following a preliminary probe selection using Wilcoxon test, the classification method ranks the probes separately for each sample; then it produces a set of subject-specific signatures, where each signature is the list of the first n1 and the last n2 probe IDs in the ranking (n1 and n2 have the same value for all subjects and are method parameters that are automatically estimated). An all-to-all signature comparison is then carried out using a distance metric based on a weighted enrichment score, resulting in a distance matrix that systematically quantifies the degree of similarity between the subjects. Subjects are finally classified by the algorithm into the two groups of APOE33 and APOE44 carriers, by assigning each sample to the group of subjects whose elements have the lowest averaged distance from the sample. In the enhanced version used here the original method was extended with a global optimizer that automatically selects the method parameters, such as signature length (n1 + n2) and feature selection stringency, using a genetic algorithm. The classification accuracy of the method has been evaluated using a ten-fold cross-validation scheme, producing an averaged accuracy on the validation set equal to 85%. Moreover, we evaluated the statistical significance of the analysis by means of a permutation test in which we compared the observed classification accuracy of the method with an empirical distribution of accuracy values obtained by 10000 random permutations of the probe labels. As a result of the permutation test we obtained a p-value ≪ 0.001. Finally, a transcriptional biomarker has been extracted by considering the 114 probes (corresponding to 108 genes) that have been included in at least one sample signature. Such a biomarker identifies the smallest subset of genes that allows the classifier to discriminate between APOE44 and APOE33 carriers.

### Network analysis

The list of genes included in the transcriptional biomarker has been computed to be the shortest possible while maintaining the highest possible classification accuracy. In order to facilitate the interpretation of the biological processes underlying the phenomenon under investigation, we better characterized the list of genes constituting the biomarker by means of a network analysis technique implemented in NetWalker^14^, a network analysis suite for functional genomics. NetWalker is a tool for identifying a high-scoring sub-network resulting from a random walk exploration over a user-provided interaction network, where the exploration takes into account the activity scores for a list of genes of interest. In our analysis, we used the protein-protein interaction network (PIN) taken from the Human Protein Reference Database (HPRD)^15^ as background network, the genes included in the biomarker as the set of genes of interest and the negative log2 of the p-values computed during the probe filtering step in the biomarker identification algorithm as gene scores. The NetWalker output consisted of a list of interactions of the background network and their scores as computed by the algorithm. In our analysis we empirically selected a relevance threshold for the interaction scores as the value corresponding to an obvious step in the graph of sorted interaction scores. This conservative selection of a threshold ensures that the scores are well above the level of random values, and that only highly significant interactions are retained. We then extracted the connected sub-network containing all interactions above the threshold (n = 504 selected interactions). The final list of genes, which integrated the one constituting the transcriptional biomarker, was given by the genes included in such a sub-network, called the “NetWalker network” in the remainder of the paper (n = 627 genes).

### Functional annotation analysis

The gene lists obtained from network enrichment analysis were used to extract the most representative GO Biological Process terms (i.e. the ones that are over-represented, but that do not refer to most general biological processes). For identifying and visualizing enriched GO terms, we used GOrilla and REVIGO tools^16^,^17^. Hypergeometric distribution was applied to test GO term enrichment, and an adjusted p-value (FDR) threshold of 0.001 was selected. As reference background the complete HPRD gene list was used. Pathway analysis was performed using ConsensusPathDB^18^. Hypergeometric distribution with an adjusted p-value (FDR) threshold of 0.001 was applied to test pathway enrichment, using HPRD gene list as reference background. GOrilla and ConsensuPathDB tools were selected because they provided reliable statistical analysis tools together with an updated version of the databases.

### Analysis of epistasis

To investigate the presence of a genetic interaction between APOE and the SNPs in genes corresponding to proteins identified in the APOE44 network we used two datasets of genotype data (see Transcriptomic and Genomic data section, studies number 4–5, Table 1). For each dataset we selected the individuals affected by Alzheimer’s disease with disease age-at-onset available and we performed a Quality Control (QC) procedure. SNPs were mapped to the gene only if they were located within the gene boundaries and they were removed if not on autosomes, if they had a call rate lower than 95% and a frequency of the minor allele less than 0.05. Samples were excluded when they had a call rate less than 95%. Furthermore, for the ADNI dataset samples were excluded if not of self-declared Caucasian origin and if outliers in multidimensional scaling (MDS) analysis. To minimize confounding due to population stratification we adjusted the analysis for the top three MDS coordinates. After the QC procedure, the ADNI study included 139 individuals and 522092 SNPs. The Mayo dataset included 580 individuals and 294128 SNPs. The drop in samples number is due to the design of our study which required a stratification based on the diagnosis of Alzheimer’s disease, availability of APOE genotype (only APOE33 and APOE44 carriers were selected) and information on the age at onset of the disease. The genetic interaction between APOE and each SNP was tested using a linear regression model that included the interaction term, as implemented in PLINK^19^. Specifically, we used the age-at-onset of Alzheimer’s disease as a response variable since APOE4 allele is associated with the age of onset of late onset Alzheimer’s disease^2^ and the APOE dosage, the SNP under investigation and the APOE-SNP interaction as explanatory variables. To minimize confounding due to population stratification we adjusted the analysis for the top three MDS coordinates. To test the presence of a genetic interaction we considered the p-value of the interaction APOE-SNP from PLINK output. The results of the two datasets were combined in a meta-analysis using METAL^20^. A fixed effects, sample size weighted approach was applied and the genomic inflation factor was used to correct for putative population stratification.

The SNPs in common between the two datasets and showing the same direction of effect in the two studies were mapped to the genes identified as APOE molecular interactors. In total, 2579 SNPs were mapped to 389 genes. We considered as significant those interactions with a q-value (Estimated False Discovery Rates as calculated with R fdrtool package) less than 0.05.

## Results

**Figure 2:**
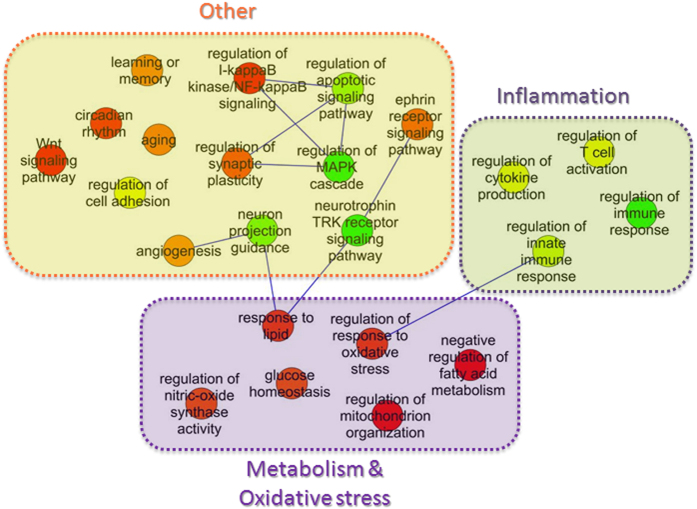
Schematic graphs of over-represented Gene Ontology biological process terms in APOE44 NetWalker network. GO terms are represented as nodes, and the strongest GO term pairwise similarities are designated as edges in the graph. GO terms are grouped to illustrate the main biological processes characterizing APOE44 subjects. Complete list of GO terms can be found in Supplementary Table S1.

**Figure 3:**
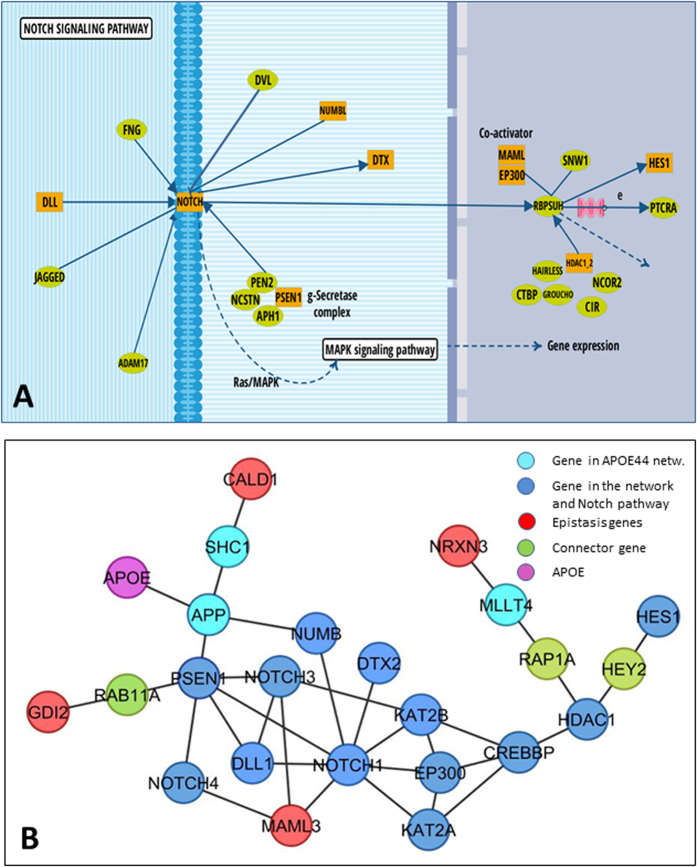
Flow diagram representing the molecular interactions in the Notch pathway (graphically adapted from KEGG database). The Notch pathway is enriched with the network proteins, labeled in orange (panel A). In panel B, the sub-network of the genes belonging to the Notch pathway and their interaction with the epistasis genes are shown.

We extracted the transcriptomic profiles of APOE33 (n = 47) and APOE44 carriers (n = 30) from the Myers’ dataset (study number 1, Table 1) and used the rank-based signature definition algorithm to identify a list of signature genes capable of discriminating the two groups with high accuracy. The resulting molecular signature consists of 114 probes corresponding to 108 transcripts and is able to classify the subjects as either APOE33 or APOE44 carriers with an accuracy of 87% (precision = 0.86, recall = 0.87, average values obtained from the cross-validation procedure). In order to identify the functional biological processes context of the transcripts includes in the identified biomarker, we carefully expanded this list following a classical weighted network based analysis. Specifically, the list of signature genes was used as input to the NetWalker tool for network analysis, using the p-values computed by the signature algorithm as weights, as described in the Materials and Methods section. The resulting network is composed of 650 proteins and it represents the set of genes and interactions on which a canonical functional enrichment analysis of GO Biological Process terms was carried out. Figure 2 summarizes the main functional annotation analysis results. This analysis revealed a predominant role of metabolic processes and oxidative stress including glucose and lipid metabolism, mitochondria and insulin-related functions. Interestingly, biological processes related to inflammation with innate immune response, regulation of cytokine and interferon functions were also represented. Other processes were evidenced comprising regulation of synaptic plasticity, regulation of neurogenesis, learning and memory, and circadian rhythm. Finally, a role of angiogenesis and regulation of programmed cell death was also evident. Pathway analysis supported also the role of inflammatory pathways evidenced in the GO term analysis including chemokine signaling. A central role of metabolic processes such as insulin pathways was also evident. In addition, several signaling pathways including WNT, Notch and FoxO were over-represented (Table 2; Supplementary Table S1). In Fig. 3 and Supplementary Figure 1, the Notch and FoxO pathways, respectively, are depicted highlighting the proteins belonging to our network. Complete list of enriched pathways with associated genes can be found in Supplementary Table S1.

In order to validate the results obtained with the first dataset, an independent transcriptomic data obtained from the AMP initiative which includes cerebral cortex RNAseq analysis of AD affected individual carrying APOE33 or APOE44 alleles were studied (study number 2, Table 1). The signature consisted of 757 transcripts corresponding to 642 gene symbols, while the network contains 650 proteins. This study, including numerous individuals and a different analytical technique compared to the first data set. namely RNA sequencing, confirmed the GO terms and pathways indicated by the initial part of the study (Table 2, Supplementary Table S2), particularly the involvement of inflammatory and metabolic pathway and the central role of the Notch pathway.

The transcriptome-wide statistical analysis of microarray data from laser dissected astrocytes of APOE4+ or APOE4- individuals affected by AD (study number 3, Table 1), regardless of disease stage, did not reveal any gene whose expression was significantly different in the two groups after the correction for multiple testing was applied. However, when testing was performed on single genes of the chemokine pathway whose relevance was suggested by our own results and by previous studies^21^, several genes appeared to be altered in APO4+ carriers (Supplementary Table S3). In particular, the chemokine pathway genes VAV3, RAC1, PIK3R5, PIK3R2, CCL21, CCL4, STAT5B, and CCR2 were significantly altered using the statistical methods reported in the original article (see method section and Supplementary Figure 2). Genome wide genotyping data from the ADNI and Mayo Clinic LOAD Risk Genetic Association studies (studies number 4–5, Table 1) were also integrated in our study. SNPs falling within the genes identified with the network analysis were evaluated for their genetic interaction with APOE in modulating the disease age-at-onset. This analysis identified 6 SNPs significantly interacting with APOE (Table 3) that are located in the sequence of 4 genes: Neurexin 3 (NRXN3), Mastermind-like receptor 3 (MAML3), GDP Dissociation Inhibitor 2 (GDI2), and Caldesmon 1 (CALD1). The SNPs within the same gene resulted highly correlated in both datasets. In particular, in ADNI dataset the NRXN3 SNP pair [rs12891137 rs766024] showed an r^2^ = 0.611 and a D′ = 0.954; the MAML3 SNP pair [rs1402669 rs2874372] showed an r^2^ = 0.761 and a D′ = 0.945. In Mayo dataset the NRXN3 SNP pair showed an r^2^ = 0.717 and a D′ = 0.954; the MAML3 SNP pair showed an r^2^ = 0.679 and a D′ = 0.905.

The significant SNPs were also evaluated for eQTL association in cerebral cortex using data in GTeX database and the SNP-gene pair rs1858446-GDI2 showed a p-value < 0.05 (effect size −0.18, p-value 0.047). The other SNPs did not show significant p-values in eQTL analysis.

Among the identified genes, MAML3 belongs to the Notch pathway, a pathway already evidenced by the transcriptomic analysis. Interestingly, also the other 3 genes in epistasis with APOE are closely related to the Notch-associated genes in the NetWalker network (Fig. 3B), indicating that the alteration at the genetic level could potentially contribute to the downstream transcriptomic perturbations.

## Discussion

One distinctive aspect of our study is the combined analysis of multiple sets of data. The advantage of this approach is that conclusions supported by multiple sources of evidence are less likely to be the result of chance or data overfitting, and less prone to population or technical biases. This type of data integration also presents some challenges due to data heterogeneity both at the annotation and numerical levels. For example, the different levels of detail in the annotation on Braak stage and Brodmann area require some additional considerations in performing a direct comparison. Conversely, the detailed annotations of post-mortem delay and age at death enabled us to quickly conclude that the samples are well matched. Crucially, we were able to overcome the data heterogeneity challenge by using a functional-level integrative analysis. Thus, the study of biological networks is a powerful tool to analyze and integrate data produced by omic technologies^22^. In the present study, network analysis has revealed many aspects of the molecular interaction structure in APOE44 AD brains, when compared to APOE33 AD brains, thus providing an insight into the underlying mechanism by which APOE4 confers risk for the development of AD.

In order to build an APOE44-AD specific network we leveraged the power of complementary approaches such as biomarker identification and network analysis, to dissect the complex mechanism underlying the interaction between APOE44 genotype and AD pathology. While biomarker identification summarizes the essential features of such a mechanism, network analysis helps in deciphering it by providing a biological context. The creation of an APOE4-AD network representative of a large cohort of human brain tissue has revealed many of the genes characterizing this allelic composition inducing an increased risk to AD. The samples cohort selected for the study were matched for age and PMI, thus we believe that these factors, which may interfere with preservation of RNA, has been properly controlled. No specific information on Braak stages of each sample has been provided, thus we cannot exclude different levels of severity of the disease. However, considering that in the cortical areas studied amyloid plaques deposition and the related glial response appear evident even in the early phases of the disease^23^, we are confident that it will not have a strong impact on the study results.

Our systems biology approach, investigating the role of APOE44 in an AD milieu, highlighted the inflammatory and immune response pathways and the processes related to oxidative stress as central mechanisms (Fig. 2). This is in line with previous studies which have focused on the role of APOE in inflammatory processes suggesting that the function of APOE is closely linked with both pro- and anti-inflammatory cytokines. The interaction between APOE and cytokine appears very complex with an intricate APOE-mediated feedback regulation of inflammatory and immune responses^24^. The modulation of inflammatory response has been shown also to be isoform-specific^25^ with a higher level of TNF-α secreted by cultured macrophage derived from APOE4 transgenic mice as compared with APOE3 mice^26^. Among the pathways related to inflammation, we highlighted the chemokine signaling pathways as involved (Supplementary Figure 2). APOE4 genotype has been demonstrated to specifically modulate astrocyte secretion of potent chemotactic agents, including CCL3, found also in our network, thus providing evidence that APOE modulation of central nervous system (CNS) innate immune response is mediated through astrocytes^21^. In view of the role of astrocyte in inflammation^27^, we further analyzed the chemokine pathway in dissected astrocyte of APOE4+ and APOE4- carrier. Among the chemokine pathway, numerous genes were shown to be altered in astrocyte in APOE4+ individuals (Supplementary Figure 2), suggesting an involvement of astrocytic APOE4 in the inflammatory cascade. It has to be noted that these genes met the significance threshold (p-value < 0.02) only when tested individually but not when the correction for multiple testing was applied, suggesting that the experimental evidence is not strong enough in the context of a conventional genome-wide search. This study also highlighted the role of oxidative stress and its association with APOE44, and also with physiological aging, supporting the notion that APOE4 is anticipating or worsening what normally occurs during the aging process. Genes in the network related to the regulation and response to oxidative stress mainly belong to the Forkhead box O (FoxO) pathway (Supplementary Table S1). As shown in Supplementary Figure 1, several genes are associated with this pathway which regulates a series of cellular processes^28^ and, particularly, are activated by oxidative signals and regulate cell proliferation and resistance to oxidative stress, playing a central role in redox signaling^29^.

FOXO1, present in our network, has been previously shown to be involved in resistance to oxidative stress via the up-regulation of antioxidant enzymes^30^. FOXO transcription factors are not only involved in response to oxidative stress but also on insulin action, both of them altered in APOE44-AD network. This is a potential integrative link between AD and insulin resistance in APOE44 carriers which is increasingly thought to contribute to Alzheimer’s disease^31^. Both insulin resistance and oxidative stress stimulate back the transcriptional activity of FoxO proteins, inducing hyperglycemia and a further increased production of reactive oxygen species (ROS) closing the vicious circle^29^.

The integration of the results of the genetic analysis with the APOE network allowed us to identify a subset of genes that interact with APOE to influence disease phenotype in a biologically meaningful context. Indeed, although the epistatic interactions do not imply per se a specific physical interaction between proteins, our approach of limiting their identification to genes belonging to the APOE network improved the biological plausibility of our findings and at the same time allowed us to focus on a limited sub-network. The present approach presents, however, some limitations mainly related to the identification of the causative SNPs and genes starting from the association results. Specifically, we cannot exclude that the SNPs we identified could tag the association of causative variants located in nearby genes different from the target ones we used as starting point of the association analysis, despite their localization within the target gene boundaries. This is a limitation of the “gene-directed” strategy we used to circumvent the inherent limitations in detecting regions of LD and their associated genes. Fine mapping of risk loci is a highly debated issue in genomics and in many cases the functional genes are hard to be identified without follow-on experiments designed ad-hoc^32^,^33^. Nevertheless, all the genes in epistasis with APOE are closely related to each other in the PIN and are associated to the genes in the canonical Notch pathway (Fig. 3B), one of the enriched pathway in the APOE44 network. In particular, among the epistatic interaction identified, one resulted of particular interest: Mastermind-like 3 (MAML3), a key element of Notch signaling which forms transcriptionally activating complexes with the intracellular domains of Notch itself. The convergence in this Notch-related sub-network (Fig. 3B) of APOE and the epistasis genes, as well as of APP and PSEN1, genes on which mutations are responsible for the familiar form of AD^34^, could give a mechanistic hypothesis on how these genetic alterations lead to the earlier onset of the disease.

A link between Notch pathways and AD pathogenesis has been previously suggested based mainly on the fact that Notch is the substrate of γ-secretase/presenilin^35^. In relation to the inflammatory processes highlighted in our study, Notch signaling, possibly altered with the upstream genetic modifications, could be a central actor in view of the supported role of Notch in modulating the microglia innate response^36^ and in promoting microglia activation^37^.

In conclusion, this systems biology study of the APOE44 role in AD evidenced a crucial role of the Notch pathway in mediating the mechanism by which APOE4 increases the risk for the development of AD.

## Additional Information

**How to cite this article**: Caberlotto, L. *et al.* Integration of transcriptomic and genomic data suggests candidate mechanisms for APOE4-mediated pathogenic action in Alzheimer’s disease. *Sci. Rep.* **6**, 32583; doi: 10.1038/srep32583 (2016).

## Supplementary Material

Supplementary Information

Supplementary Table S1

Supplementary Table S2

Supplementary Table S3

## Figures and Tables

**Table 1:** Datasets used in the study.

Study	Type of data/Technology	#Subjects in the original study	#Subjects selected for the study and APOE genotype	Brain Region	Data source
1-Mayer’s lab	Transcriptomic/Illumina ref-seq 8	364	77 (43APOE33 34 APOE44)	Temporal, Parietal, Frontal Cortex	NCBI GEO GSE15222
2-Mayo	Transcriptomic/Illumina HiSeq 2000	278	39 (30 APOE33 9 APOE44)	Temporal Cortex	AMP AD knowledge Portal - syn3163039
3- Astrocyte study	Transcriptomic/2.0 Affymetrix gene arrays	18	18 (9 APOE4-9 APOE4+)	Frontal Cortex (Astrocyte)	NCBI GEO GSE29652
4-ADNI	Genomic/ILLUMINA Human610-Quad BeadChip	757	139 (APOE4: 0 = 45;1 = 65;2 = 29)	NA	adni.loni.usc.edu
5-Mayo LOAD GWAS	Genomic/ILLUMINA HumanHap300	2099	580 (APOE4: 0 = 175; 1 = 289; 2 = 98)	NA	AMP AD Knowledge Portal - syn3157238

Table describing the datasets used in the study, type of data and technology platform used, total number of subjects in the original study and number of subjects selected for the present study including APOE alleles composition. Brain regions analyzed in the study (when applicable) and data source are also described.

**Table 2:** Pathways enrichment analysis.

Pathway	Database	q-value Study #1	q-value Study #2
Signaling by Interleukins	Reactome	3.08E-19	19.36E-08
Cytokine Signaling in Immune system	Reactome	3.64E-18	4.37E-07
Neurotrophin signaling pathway - Homo sapiens (human)	KEGG	2.09E-18	4.13E-07
Innate Immune System	Reactome	5.41E-17	7.65E-07
PI3K-Akt signaling pathway - Homo sapiens (human)	KEGG	7.41E-15	1.43E-05
MAPK family signaling cascades	Reactome	1.15E-13	1.6E-06
Signaling by Leptin	Reactome	2.57E-13	6.71E-05
IGF1R signaling cascade	Reactome	1.14E-12	0.000458
Signaling by Insulin receptor	Reactome	3.65E-11	0.000785
FoxO signaling pathway - Homo sapiens (human)	KEGG	6.41E-09	5.33E-06
NF-kappa B signaling pathway - Homo sapiens (human)	KEGG	6.66E-07	>0.01
Sphingolipid signaling pathway - Homo sapiens (human)	KEGG	1.24E-06	9.67E-05
Toll-Like Receptors Cascades	Reactome	2.15E-06	0.00095
Insulin signaling pathway - Homo sapiens (human)	KEGG	7.57E-06	0.000123
Notch signaling pathway - Homo sapiens (human)	KEGG	1.86E-05	0.0016
Chemokine signaling pathway - Homo sapiens (human)	KEGG	2.54-E05	0.001149
Intrinsic Pathway for Apoptosis	Reactome	0.0011	0.005454

Table describing the most relevant pathways (with the corresponding database) found in the study and the q-value of the two independent studies (studies number 1 and 2, Table 1).

**Table 3:** Results of the analysis of a genetic interaction between *APOE* and the SNPs in genes corresponding to proteins in the APOE44-AD network.

Official Gene symbol	SNP	SNP position (hg38)	A1	A2	N	Z score	Direction of effect	p-value	q-value
NRXN3	rs12891137	chr14:79157374	T	C	718	−4.178	—	2.94 × 10^−5^	0.016
MAML3	rs1402669	chr4: 140064751	A	G	719	3.832	++	1.27 × 10^−4^	0.035
MAML3	rs2874372	chr4: 140071968	T	C	718	3.606	++	3.11 × 10^−4^	0.045
GDI2	rs1858446	chr10: 5810209	T	C	715	−3.468	—	5.24 × 10^−4^	0.049
CALD1	rs10488462	chr7: 134825047	A	G	714	3.438	++	5.85 × 10^−4^	0.049
NRXN3	rs766024	chr14: 79170051	T	C	719	3.417	++	6.33 × 10^−4^	0.050

The genetic variants located in genes belonging to the APOE network that shows a significant statistical interaction with APOE are shown. For each SNP is reported the Official Gene Symbol of the corresponding gene, the reference SNP ID, the genomic position, allele1, allele2, number of subjects, Z score, direction of effect, p-value and FDR corrected q-value.
