# Supplementary material for: Integration of transcriptomic and genomic data suggests candidate mechanisms for APOE4-mediated pathogenic action in Alzheimer’s disease
Source: Sci Rep. 2016 Sep 2;6:32583. doi: 10.1038/srep32583 (PMC5009326; doi:10.1038/srep32583)
Supplement: Supplementary Information [file srep32583-s1.pdf]

Integration of transcriptomic and genomic data suggests candidate mechanisms for APOE4-mediated pathogenic action in Alzheimer's disease.

Laura Caberlotto, Luca Marchetti, Mario Lauria, Marco Scotti, and Silvia Parolo.

Supplementary Figure 1: Flow diagram representing the molecular interactions in the Chemokine signaling pathway (modified from KEGG database using bStyle package [34]). The pathway is enriched with the network proteins, labeled in orange. Highlighted with the dotted line the genes altered in astrocytes of APOE4+ carriers.

Supplementary Figure 2. Flow diagram representing the molecular interactions in the FoxO signaling pathway (modified from KEGG database using bStyle package [34]). The FoxO pathway is enriched with the network proteins, labeled in orange.

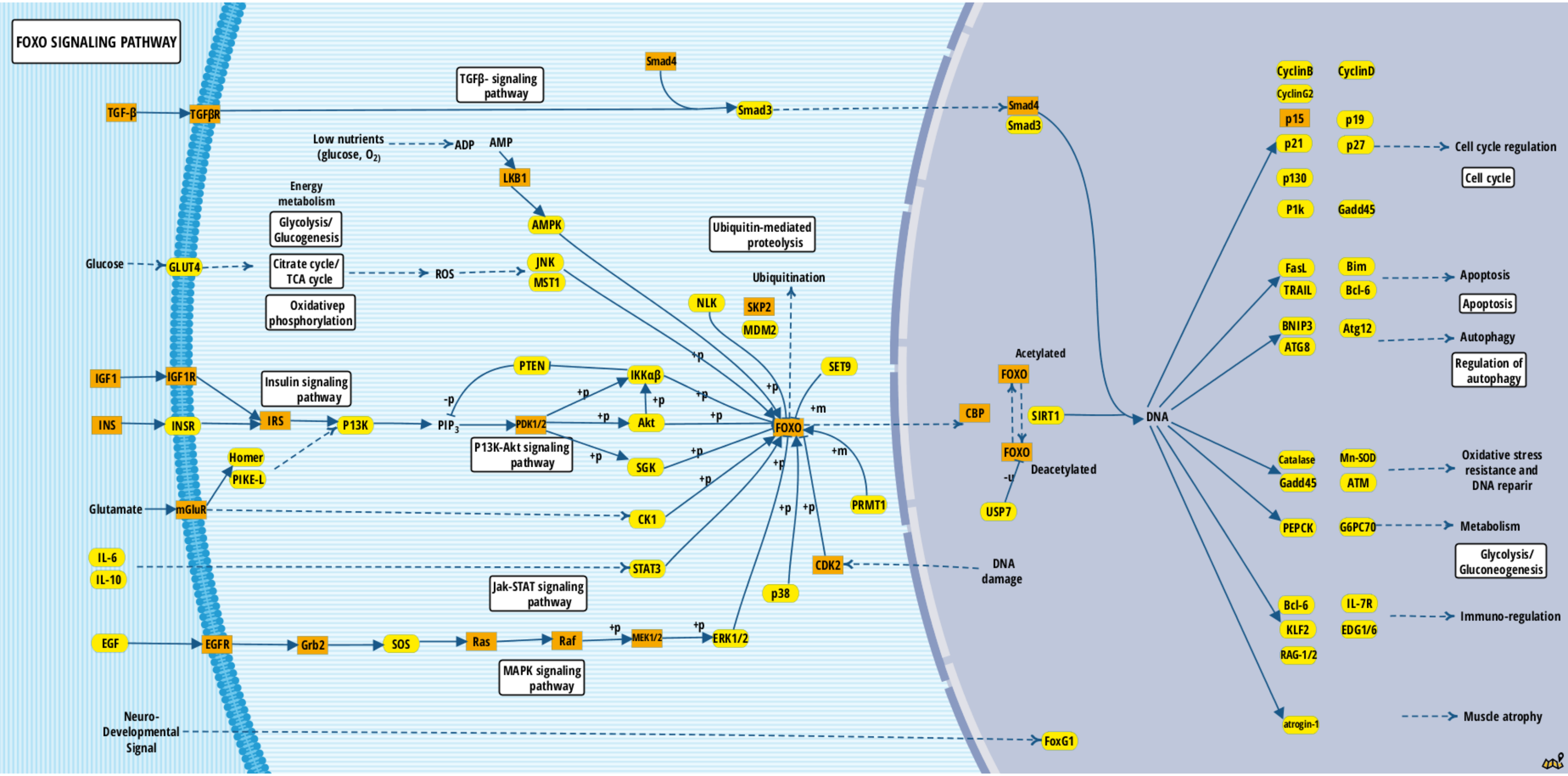
FOXO SIGNALING PATHWAY
TGF-β
TGFβR
TGFβ- signaling pathway
Smad4
Smad3
Smad4
Smad3
Low nutrients (glucose, O2)
ADP
AMP
LKB1
AMPK
Energy metabolism
Glycolysis/ Glucogenesis
Citrate cycle/ TCA cycle
Oxidativep phosphorylation
Glucose
GLUT4
ROS
JNK
MST1
Ubiquitin-mediated proteolysis
Ubiquitination
NLK
SKP2
MDM2
SET9
IGF1
IGF1R
INS
INSR
IRS
Insulin signaling pathway
P13K
PTEN
-p
PIP3
IKKαβ
+p
PDK1/2
Akt
SGK
P13K-Akt signaling pathway
Homer
PIKE-L
Glutamate
mGluR
CK1
IL-6
IL-10
STAT3
Jak-STAT signaling pathway
FOXO
+m
PRMT1
CDK2
p38
DNA damage
EGF
EGFR
Grb2
SOS
Ras
Raf
MEK1/2
ERK1/2
MAPK signaling pathway
Neuro-Developmental Signal
FoxG1
CBP
Acetylated
FOXO
SIRT1
FOXO
Deacetylated
-u
USP7
DNA
CyclinB
CyclinD
CyclinG2
p15
p19
p21
p27
Cell cycle regulation
Cell cycle
p130
P1k
Gadd45
FasL
Bim
TRAIL
Bcl-6
Apoptosis
Apoptosis
BNIP3
Atg12
ATG8
Autophagy
Regulation of autophagy
Catalase
Mn-SOD
Gadd45
ATM
Oxidative stress resistance and DNA repair
PEPCK
G6PC70
Metabolism
Glycolysis/ Gluconeogenesis
Bcl-6
IL-7R
KLF2
EDG1/6
RAG-1/2
Immuno-regulation
atrogin-1
Muscle atrophy

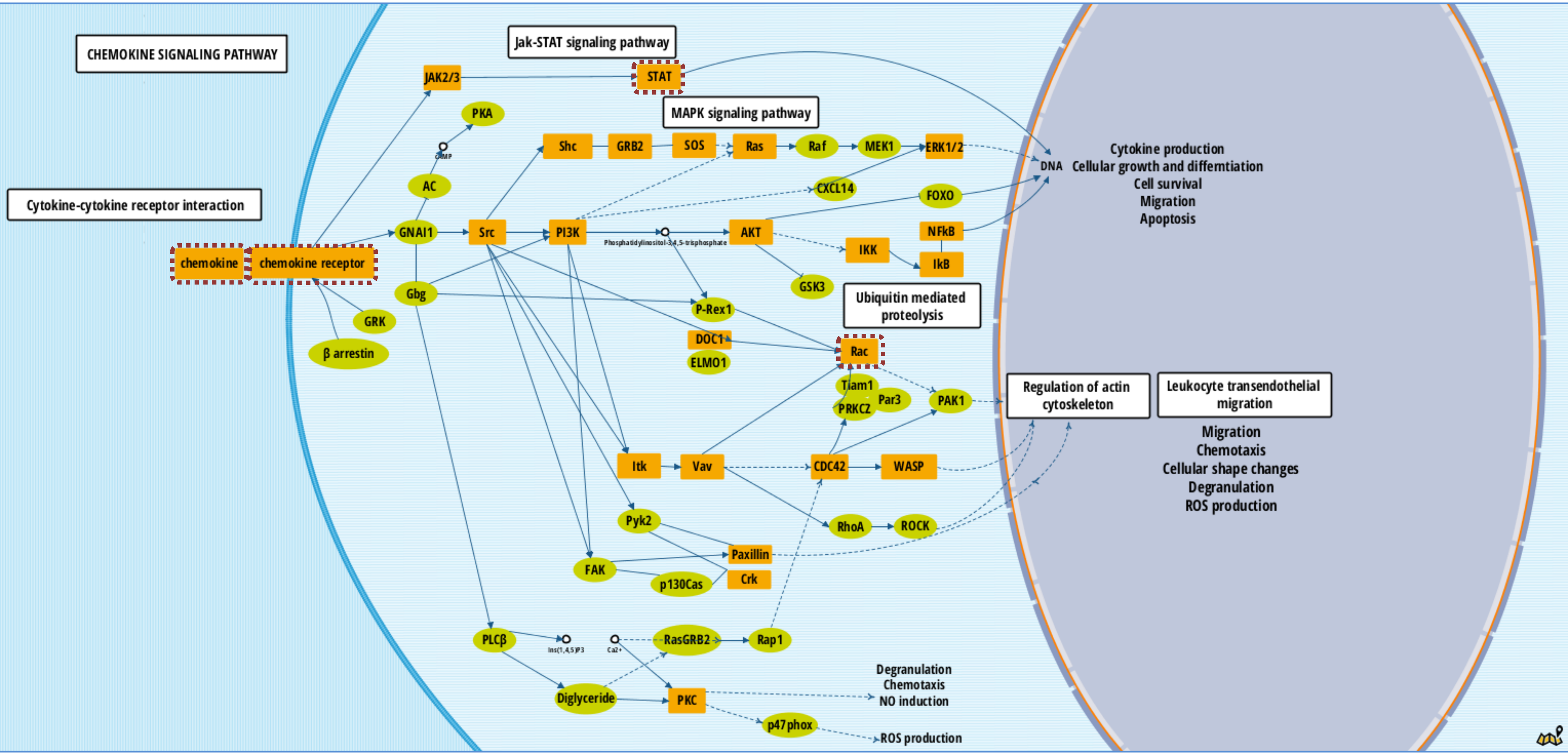
CHEMOKINE SIGNALING PATHWAY
Jak-STAT signaling pathway
MAPK signaling pathway
Cytokine-cytokine receptor interaction
Ubiquitin mediated proteolysis
Regulation of actin cytoskeleton
Leukocyte transendothelial migration
chemokine
chemokine receptor
GRK
β arrestin
JAK2/3
STAT
PKA
cAMP
AC
GNAI1
Gbg
Src
Shc
GRB2
SOS
Ras
Raf
MEK1
ERK1/2
CXCL14
FOXO
PI3K
Phosphatidylinositol-3,4,5-trisphosphate
AKT
IKK
NFkB
IkB
GSK3
P-Rex1
DOC1
ELMO1
Rac
Tiam1
Par3
PRKCZ
PAK1
Itk
Vav
CDC42
WASP
RhoA
ROCK
Pyk2
Paxillin
Crk
FAK
p130Cas
PLCβ
Ins(1,4,5)P3
Ca2+
RasGRB2
Rap1
Diglyceride
PKC
p47phox
DNA
Cytokine production
Cellular growth and differntiation
Cell survival
Migration
Apoptosis
Migration
Chemotaxis
Cellular shape changes
Degranulation
ROS production
Degranulation
Chemotaxis
NO induction
ROS production
